# Performance-based incentives may be appropriate to address challenges to delivery of prevention of vertical transmission of HIV services in rural Mozambique: a qualitative investigation

**DOI:** 10.1186/s12960-016-0157-0

**Published:** 2016-10-07

**Authors:** Roseanne C. Schuster, Octávio de Sousa, Jacqueline Rivera, Rebecca Olson, Delphine Pinault, Sera L. Young

**Affiliations:** 1 Program in International Nutrition, Division of Nutritional Sciences, Cornell University, Ithaca, NY 14853 United States of America; 2 School of Human Evolution and Social Change, Arizona State University, Tempe, AZ 85287-2402 United States of America; 3 CARE Mozambique, 596 Av. Mártires de Mueda, Maputo, Mozambique; 4 Division of Nutritional Sciences, Cornell University, Ithaca, NY 14853 United States of America; 5 Humphrey School of Public Affairs, University of Minnesota, 310 19th Street S, Minneapolis, MN 55455 United States of America; 6 CARE Uganda, CARE Mozambique, 596 Av. Mártires de Mueda, Maputo, Mozambique; 7 Program in International Nutrition, Department of Population Medicine and Diagnostic Sciences, Cornell University, Ithaca, NY 14853 United States of America; 8 Department of Anthropology, Northwestern University, 515 Clark Street, 60208 Evanston, IL United States of America

**Keywords:** Prevention of mother-to-child transmission of HIV, Performance-based incentives, Maternal and child health nurses, Community health workers, Traditional birth attendants, Motivation, Health systems, Mozambique

## Abstract

**Background:**

Performance-based incentives (PBIs) have garnered global attention as a promising strategy to improve healthcare delivery to vulnerable populations. However, literature gaps in the context in which an intervention is implemented and how the PBIs were developed exist. Therefore, we (1) characterized the barriers and promoters to prevention of vertical transmission of HIV (PVT) service delivery in rural Mozambique, where the vertical transmission rate is 12 %, and (2) assessed the appropriateness for a PBI’s intervention and application to PVT.

**Methods:**

We conducted 24 semi-structured interviews with nurses, volunteers, community health workers, and traditional birth attendants about the barriers and promoters they experienced delivering PVT services. We then explored emergent themes in subsequent focus group discussions (*n* = 7, total participants *N* = 92) and elicited participant perspectives on PBIs. The ecological motivation-opportunity-ability framework guided our iterative data collection and thematic analysis processes.

**Results:**

The interviews revealed that while all health worker cadres were motivated intrinsically and by social recognition, they were dissatisfied with low and late remuneration. Facility-based staff were challenged by factors across the rest of the ecological levels, primarily in the opportunity domain, including the following: poor referral and record systems (work mandate), high workload, stock-outs, poor infrastructure (facility environment), and delays in obtaining patient results and donor payment discrepancies (administrative). Community-based cadres’ opportunity challenges included lack of supplies, distance (work environment), lack of incorporation into the health system (administration), and ability challenges of incorrect knowledge (health worker). PBIs based on social recognition and that enable action on intrinsic motivation through training, supervision, and collaboration were thought to have the most potential for targeting improvements in record and referral systems and better integrating community-based health workers into the health system. Concerns about the implementation of incentives included neglect of non-incentivized tasks and distorted motivation among colleagues.

**Conclusions:**

We found that highly motivated health workers encountered severe opportunity challenges in their PVT mandate. PBIs have the potential to address key barriers that facility- and community-based health workers encounter when delivering PVT services, specifically by building upon existing intrinsic motivation and leveraging highly valued social recognition. We recommend a controlled intervention to monitor incentives’ effects on worker motivation and non-incentivized tasks to generate insights about the feasibility of PBIs to improve the delivery of PVT services.

**Electronic supplementary material:**

The online version of this article (doi:10.1186/s12960-016-0157-0) contains supplementary material, which is available to authorized users.

## Background

The implementation of evidence-based biomedical practices and policies over the past decade has demonstrated potential to significantly reduce rates of vertical transmission of HIV to as low as 1–2 % [1]. The continuum of care to prevent vertical transmission of HIV (PVT) includes maternal HIV testing, prenatal and postnatal antiretroviral therapy (ART) and prophylaxis, safe birth practices, safe infant and young child feeding, and early infant HIV testing [2]. However, barriers to this cascade contributed to 199 000 infants and young children becoming HIV infected in sub-Saharan Africa in 2013 [3]. There, HIV-infected women experience individual, family, community, health system, and structural barriers that result in their drop-off from each step in the continuum of PVT care [4, 5]. Simultaneously, health workers face challenges that affect their motivation, opportunity, and ability to deliver PVT services [6].

Mozambique experiences significant challenges to both uptake and delivery of PVT services. First, women of reproductive age experience a high prevalence of HIV (16 %), fifth-highest among women aged 15–24 globally [7]. Second, despite significant progress over the past decade, coverage of PVT services remains low: only 42 % of pregnant women received HIV counseling, testing, and test results during antenatal care [8], and 35 % of HIV-exposed infants were HIV tested by 2 months [7]. These drop-offs, and sub-optimal coverage of other PVT services, resulted in 12 % of HIV-exposed Mozambican children becoming infected with HIV in 2013 [9]. Finally, both facility- and community-based health workers deliver PVT services in Mozambique, reflecting the widespread trend of “task shifting,” or movement of tasks from more to less specialized health workers, in HIV care.

Performance-based incentives (PBIs) have been effective in improving uptake and delivery of health services in low-resource settings. On the delivery side, PBIs are the distribution of money or material goods after a performance target has been achieved and aim to counteract weak health system incentives by aligning rewards with health outcomes [10]. Delivery-focused PBIs have increased institutional births and antenatal care attendance, preventative child visits, and HIV testing in African nations including Rwanda, Nigeria, Tanzania, and Benin [10–15]. A situational analysis recognized PBIs as appropriate and feasible in Mozambique and suggested that PBIs could support task shifting, motivate community health workers, and mitigate health facility challenges to service delivery [16]. Indeed, PBIs were successfully used to achieve process improvements in the Mozambican medical supply chain [17]. However, until recently, PBIs had been underinvestigated in the context of PVT [18].

Therefore, we (1) characterized the barriers and promoters experienced by health workers delivering PVT services in rural Mozambique and (2) assessed the potential for PBIs to support delivery of PVT services.

## Methods

### Theoretical underpinnings

**Fig. 1:**
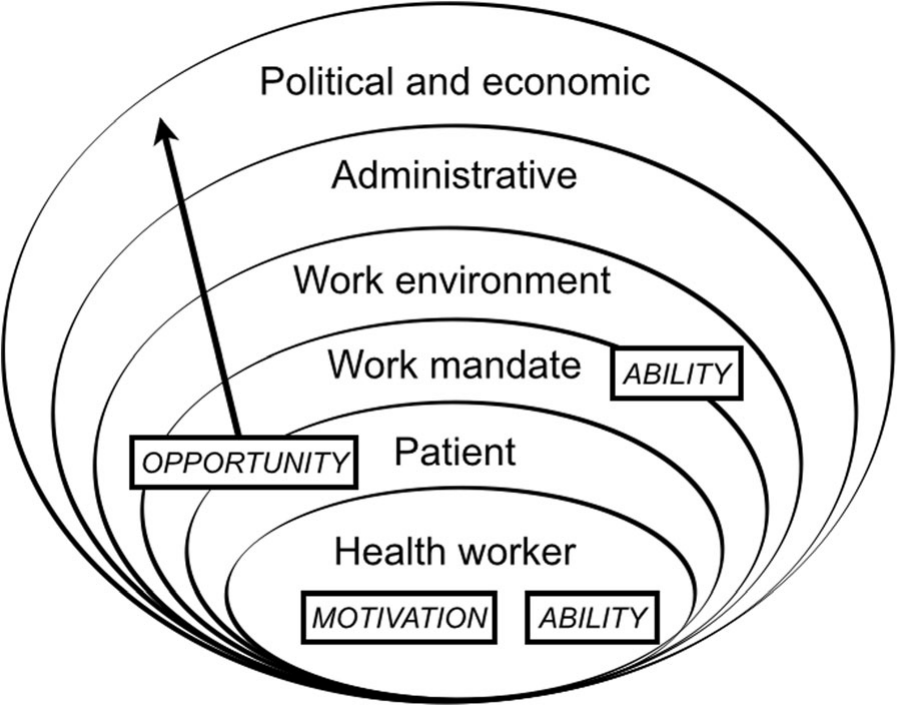
The integrated ecological motivation-opportunity-ability framework for health workers delivering prevention of vertical transmission of HIV services, modified from [6] p. 785

Two complimentary frameworks were applied to guide the study design and data analysis [6] (Fig. 1). The first was the ecologically embedded determinants of performance research agenda, which is embedded within an ecological framework that places the health worker at the center and moves outwards towards the political and economic environment [19]. The second was the motivation-opportunity-ability framework, which is grounded in human resources and operations management [20] and posits that three domains are required for optimal worker performance [21]. In integrating these frameworks, we operationalized “motivation” as the individual’s desire and willingness to act. “Opportunity” encompassed the many contextual factors that enable action beyond the individual. “Ability” included the skills and knowledge to execute action and overlaps with both the individual level and more distal levels of the ecological determinants of performance framework [6].

### Study setting

Mozambique’s legacy of colonization, war for independence (1964–1974), and civil war (1977–1992) left its health system and infrastructure unprepared for the HIV/AIDS epidemic [22]. Today, Mozambique has only 4 physicians and 41 nurses per 100 000 people, far below the regional average [23]. Task-shifting initiatives such as training mid-level *técnicos de cirúrgia* (surgical technicians) have helped address the skilled labor shortage for surgical needs [24] but have not alleviated the workload of nurses and midwives who deliver PVT services, which were integrated into antenatal care at the primary care level and free to patients [25].

We conducted this research in 2012 in a rural district in northern Inhambane Province, where CARE International was the PEPFAR-implementing partner. The district had a population of 56 000, few maintained roads, and irregular public transportation. In 2012, there were approximately 2700 pregnancies in the district, with an estimated 53 % of births occurring at health facilities [26]. HIV prevalence among pregnant women attending antenatal care was 10.5 % [27].

The public health system was comprised of one type III health facility in the district capital, one type III peripheral health facility, and four type II peripheral health facilities. The two physicians for the entire district were based at the type III facility in the district capital. A *técnico* led the largest peripheral health facility and nurses led the others. At the time of the study, the district and large peripheral type III facilities were the only facilities where patients could access ART (when CD4 count ≤350 cells/mm); only antiretroviral prophylaxis was available at the type II peripheral facilities. There were no private health facilities or physicians. A number of *curandeiros* (traditional healers) practiced in the district.

Four cadres of health workers provided PVT services within the district (Table 1). Maternal and child health nurses provided the majority of clinical PVT services at health facilities. *Activistas*, or community volunteers, provided home care and counseling to individuals living with HIV/AIDS and received supervision and financial support from CARE International. Community health workers (CHWs) provided a broad portfolio of health services to households within 10 km^2^ of their home [28]. CHWs were trained in late 2011 and began working in early 2012 and received support and supervision from the implementing partner Malaria Consortium in addition to the Ministry of Health [29]. Traditional birth attendants (TBAs) historically assisted with home births but now increasingly focused on referral for health facility deliveries. TBAs were not systematically organized or supervised.

**Table 1:** Organizational structure and description of key services provided by the four health worker cadres preventing vertical transmission of HIV in rural Mozambique

Cadre	Key services	Organization	Receives supervision	Salary	Supported by
Maternal and child health nurses	Antenatal care, birth, children 0–5 years, family planning, and clinical PVT services	Health facilities	Yes	US$ 250/month	Ministry of Health, CARE International via PEPFAR
*Activistas* (community volunteers)	Home-based care, counseling (e.g., treatment adherence, appointment follow-up), linking HIV-infected individuals to clinical care	Association (3 leaders, 20 *activistas*)	Yes	850 MZN/month (~US$ 28)	CARE International
Community health workers (CHWs)	Portfolio of health promotion and preventative care (e.g., sanitation and hygiene, malaria, and maternal and child health including breastfeeding and family planning, limited HIV/AIDS), limited curative care	Reports to coordinator at health facility	Yes	1 200 MZN/month (~US$ 40)^a^	Ministry of Health and Malaria Consortium
Traditional birth attendants (TBAs)	Refer women to health facilities for antenatal care and delivery and occasionally assist in home births	Not formally organized	No	Uncompensated	N/A

^a^60 % of the minimum monthly salary, per government recommendations

### Semi-structured interviews

#### Participant selection

To characterize health workers’ barriers and promoters (objective 1), we recruited members of the four cadres for semi-structured interviews. Maternal and child health nurses were purposively sampled based on their role and type of health facility (district, large peripheral, small peripheral). Key informants from two *activista* associations identified *activistas*, who were purposively sampled based on level of engagement. CHWs were identified by their district coordinator and were invited to participate when they visited the health facility to stock-up on supplies. A convenience sample of TBAs attending a training jointly facilitated by the district health authority and CARE International was invited to participate. Sample sizes for each cadre were based upon achieving the saturation needed to outline overarching themes [30], with the intent to expound upon these in subsequent focus group discussions.

#### Data collection

The interview guide contained questions about participants’ experiences delivering care to HIV-infected women and their HIV-exposed children, as well as their perceptions of the barriers and facilitators mothers face in the uptake of PVT services (Additional File 1). The guide was modified for each health worker cadre and pre-tested with the corresponding cadres in a neighboring district.

Two Mozambican research assistants experienced in qualitative research conducted the interviews from September 2012 to January 2013. Interviews were conducted in Xitswa or Portuguese per participant preference and took approximately 60 min. Participants were interviewed in private spaces at health facilities (nurses, *activistas*), in their communities (CHWs), and training site (TBAs).

### Focus group discussions

#### Participant selection

To share, validate, and expound upon early findings from the interviews (objective 1) and assess the appropriateness of PBIs for PVT service delivery (objective 2), *activistas*, TBAs, and an array of facility-based health workers were recruited for focus group discussions. Representatives of each health sector at the district and large peripheral facilities and all staff at the small peripheral health facility were invited to participate because key informants strongly felt that all staff contributed to PVT care. All members of the two *activista* associations were invited to participate. TBAs known to be active in their communities were invited through key informants and snowball recruitment. No focus group was conducted with CHWs because concurrent interviews with HIV-infected mothers did not show women were receiving PVT services from them at that time.

#### Data collection

We conducted a total of seven focus groups lasting 90–120 min in March 2013. Participants were asked about types of incentives, how goals should be set and assessed, and concerns about implementing PBIs. One focus group was conducted at each of the three health facilities (district type III facility (*n* = 12); peripheral type III facility (*n* = 12); type II (*n* = 3)) in Portuguese. One focus group was conducted with each of the two *activista* associations in Xitswa at their respective meeting locations (*n* = 22 each). A sixth focus group with TBAs (*n* = 6) was conducted in Xitswa at a community meeting location. The final focus group was conducted with representatives from each of the three health facilities and two *activista* associations (total *n* = 13).

### Participant observation

The interviews and focus groups were complimented by participant observation primarily conducted at the three health facilities and at *activista* meetings between July 2012 and March 2013. Participant observation at the two type III and one type II facilities was conducted at minimum on a biweekly basis at each facility, during facility business hours, and included a few facility-wide meetings on strategies for integrated HIV/AIDS case management. Participant observation at *activista* association meetings was conducted once per month. Handwritten notes were recorded and were subsequently typed.

### Analysis

The interviews and focus groups were audio recorded and accompanied by detailed handwritten notes. The interviews were transcribed into Portuguese, and the detailed notes from the focus groups were typed in Portuguese with the support of the audio recording. All transcripts were translated into English and were coded by two co-authors using the thematic analysis approach [31]. Interview results were shared with focus group participants to prompt further discussion, creating an iterative analysis process. Participant observation data was used to triangulate themes and validate findings [32].

## Results

### Roles in the context of PVT

Of the 24 health workers who participated in interviews (Table 2), nurses and *activistas* reported the greatest involvement in PVT. One nurse summarized, “My role is to counsel an HIV-infected woman in a way that she will understand that even if she has HIV, the baby can be born without the virus if she follows the recommendations that we give her” (Female nurse, 3 years of experience). *Activistas* provided in-depth counseling to pregnant women on treatment adherence, inclusive of checking pill bottles and reviewing appointment schedules, and counseled on infant and young child feeding and family planning. CHWs reported that HIV services were a small component of their portfolio and mainly advised uptake facility-based care, adherence to treatment regimens, infant feeding, and family planning. All TBAs advised women on family planning, and most advised on breastfeeding, HIV testing, and uptake of prenatal and postnatal healthcare. TBAs saw themselves in a unique position to broker resistance to health facility delivery by accompanying mothers.

**Table 2:** Characteristics of the Mozambican health workers who participated in semi-structured interviews, by cadre (*n* = 24)

	Facility-based	Community-based		
Characteristics	Maternal and child health nurses (*n* = 4)	*Activistas* (*n* = 6)	Community health workers (*n* = 6)	Traditional birth attendants (*n* = 8)
No. female	4	2	2	8
Reason for taking up role	Passion for helping women and children	Own HIV diagnosis; death of loved one due to HIV/AIDS; remote location	Chosen by community, previous work as community health workers	Assisted woman or self in childbirth; worked with facility-based workers
Mean no. years in role or similar (range)	3 (1–7)	3.5 (3–4)	10 (1–21)	16.3 (9–33)
Length of initial formal training	20 months	2 weeks	4 months	Not standard
Frequency of refresher trainings	At least annually	Annually	Annually	Very irregularly
Avg. no. patients/day (range)	26 (10–40)	3.8 (2–5)	11 (6–15)	^a^
No. has another job	0	6	4	8

^a^Traditional birth attendants were unable to quantify their average patient number on a daily, weekly, or monthly basis due to great variability

### Characterizing barriers and promoters experienced by health workers (objective 1)

Detailed results were organized according to the integrated ecological motivation-opportunity-ability framework, starting with the most proximal ecological level within each motivation, opportunity, and ability domain (Table 3). Key findings are summarized in the text below.

**Table 3:** Barriers and promoters to delivering prevention of vertical transmission of HIV (PVT) services according to the ecological motivation-opportunity-ability (MOA) framework

Barrier (−), promoter (+)	Construct	Manifestation	Maternal and child health nurses, with other health facility workers from focus group discussions (FGDs)	*Activistas*	Community health workers (CHWs)	Traditional birth attendants (TBAs)
Motivation						
Health worker factors						
	Intrinsic motivation					
+		Highly intrinsically motivated	“…when I help the community. It is what my heart wants” (F Nurse, 7 years)	“in the community there are no longer many people left living with HIV and without treatment…I feel happy that I attended to and helped [these] people” (M *activista*, 4 years)	“I help the people and my family as well. When health problems arise in my house I can resolve the problems more easily” (M CHW, 20 years)	“I never worked because I wanted money. I first worked because I wanted to help the community and then because I wanted to learn” (TBA, 12 years)
−		Dissatisfied when unable to help patients	“I do not like when difficult situations arise that I cannot solve….There was a case of a woman who had complications in birth. We tried to help but could not. The woman ended up dying” (F nurse, 3 years)	“A negative experience that struck me was a woman who refused to go to hospital and when her son died three weeks after birth” (F *activista*, 4 years)	“After some time, the person left [treatment] and the disease worsened…I counseled and the person agreed to go to the hospital…but lost [his/her] life” (F CHW, 21 year)	Women “refuse” to go to the health facility for delivery, placing TBAs in a compromised position (TBA, 17 years)
	Extrinsic motivation					
+		Recognition from patients	“…when people appreciate my work. There was a case of a women that came up to me in [] and told me that I had saved her life. I did not even remember her” (F nurse, 3 years)	Good relationship with patients	“The people that I help always thank me and respect me” (M CHW, 2 years)	“The fact that I was chosen by the pregnant women to help already shows that the woman has trust in me. And I try to live up to this trust” (TBA, 9 years)
±		Recognition from community	“The people acknowledge us” (F nurse, 3 years)	“This patient insulted me, told me to leave, but I did not give up and ended up taking him to the hospital …he ended up dying. Today [at the funeral]…people said that I helped alot when the patient was sick” (M *activista*, 4 years)	“When the person died the family … said I …forced the person to go to the hospital, while the family wanted to take him to the healers” (F CHW, 21 year)	“I like to work because it gives me prestige in the community. I have recognition in my community. And people in my community trust me” (TBA, 33 years)
±		Recognition from other health worker cadres	-	Felt that their work linking patients to care was not valued by facility staff	Concerned that patient non-adherence reflects poorly on their work quality	“When a lot of time has passed between my presence at the health center, they miss me and ask where I have been” (TBA, 20 year)
±		Role identification	Data analyst and lay counselors did not have identification badges to signify their roles at health center (FGD participants)	Want T-shirts, hats, identification cards for work in the community	Had bright green vests	Not always recognized as a TBA when at health facility
−		Patient complaints	“People always criticize our actions. The people say that they are not attended to well. It is complicated when you always receive criticism” (F nurse, 7 years)	Poor reception in homes (*activistas* insulted), patient complains *activista* has not visited often enough	Sick adults questioned why CHW cannot dispense medication to adults (e.g., for malaria)	-
−		Dissatisfaction with remuneration	Salary was low for amount of time spent (including late evenings, weekends); “Wages always arrive late” (F nurse, 3 years)	Subsidy spent on transportation to visit patients and attend meetings, to fix bicycles: “When the 850MT arrives, we have a lot of debt” (F *activista*, 3 years)	Subsidy had been delayed several months but was paid at time of interviews	“A little financial help would be really good, because we work but we do not receive anything” (TBA, 12 years)
Opportunity						
Patient factors						
−	Patient behavior circumventing care		“[Retaining women in PVT care] is a big war, because some agree to take the [HIV] test, follow the treatment during their pregnancy, but after the birth…the mother prefers to follow-up for the child and the mother abandons the treatment [for herself]” (F nurse, 7 years)	HIV-infected patient gave wrong address to reception so *activista* will not be seen at their house	-	“Many women say that they have yet to be full term and then have the baby at home” (TBA 443) TBAs feel obligated when called to assist woman in home delivery, even though they know it is against policy
Work mandate factors						
−	Referral systems		Mothers were referred to type III facilities for ART without follow-up	Patients returned to care remain on referral list given to *activistas, who* sometimes received two different referrals for same patient	-	No formal referral system; concerned about breaking confidentiality if refer a mother for ANC before mother is ready for pregnancy to be public
−	Record systems		Paper records for PVT (digital only for patients on ART); multiple nurses have to share one book (PO); problematic implementation of mothers’ PVT codes (FGD)	*Activista* leader compiled 20 *activista *members' visits and services delivered by hand to generate reports	-	Newly implemented system: women reported facilitydelivery to her local TBA, who recorded information in a notebook and shared with community leader
Work environment						
±	Workload		“It is hard because we are few; when I am attending to a person and other people are complaining about the wait outside. We do the most we can, but it is a lot of things to do” (F nurse, 7 years)	Time spent was appropriate for volunteer position	Majority felt time spent was appropriate; however, sometimes patients came to their home which interrupted their household duties	-
−	Supplies		“We always have a lack of medicines” (F nurse, 3 years)	Lacked items for conducting home care (e.g., comb, bucket, soap, gloves)	Wanted gloves, scissors, medications for treating adults	Lacked materials to attend to “emergency” birth (e.g., gloves, mask, scissors, gown), flashlight for nighttime travel
−	Infrastructure		Lack of privacy (e.g., child consultations conducted in open air hallways, lack of screens for women in maternity ward), lack of electricity and locks	Lack of office space (for meetings and storage) and equipment (e.g., desk, computer) for report writing	-	-
−	Distance		-	“What is difficult are the long distances that run between the houses and [being] without any means of transport” (M *activista*, 4 years); had bicycles but many now broken and no funds for upkeep	Distance manageable with bicycles but sandy paths were challenging	“It is a long distance to arrive at the health facility….the homes are far away from each other” (TBA 448)
Administrative environment						
	PVT service planning and coordination					
+		Efforts to streamline care	Implementation of streamlined “one-stop” care and Option B+ (lifelong ART)	-	-	-
−		Processes create delays	Long waits for results for CD4 count (7 days), PCR (up to 6 months) due to analysis at regional and national hospitals; type II peripheral facility did not offer ART	-	-	-
−		Lack of incorporation into health system		Individual work linking patients to clinical care not recorded at health facility	-	“The women living with HIV, generally they go to the facility but when they…abandon care, I don’t know [about it]” (TBA, 12 years). TBAs’ interactions with health facilities and activities varied widely
	Donors					
−		Financial practices differ from local standards	HIV-specific facility-based staff supported directly by PEPFAR (e.g., data analyst) did not receive raise when others did	-	-	N/A
Ability						
Health worker factors						
	Health worker approach to patient interactions					
+		Sensitive to patients’ opportunity challenges	“We sensitize [newly diagnosed HIV-infected woman] that if she does not have the courage to talk wither her husband, we can help” (F nurse, 7 years)	Returned to counsel and care for patients even when verbally abused; encourage feeding complementary foods from farm	Report women experience food insecurity, cost of travel to health center	Discussing family planning: “They see the cost of living each day…if you have a lot of children everything is expensive and it isn’t easy to sustain many children…the women don’t manage to feed themselves regularly.” (TBA, 7 years)
−		Language barrier	One nurse did not know local language at her first posting	-	-	-
	Knowledge					
±		Infant and young child feeding (IYCF)	Correct, updated IYCF messages	Mix of correct and incorrect IYCF messages for HIV-infected women	Correct IYCF messages for HIV-uninfected women	Incorrect, outdated IYCF messages for HIV-infected and HIV-uninfected mothers
Work environment						
±	Training		“It would be helpful for all colleagues to have the same capacity” (F nurse, 7 years); logistical challenges to training all nurses at once, potential for weariness	-	“There are cases of people who abandon [HIV] care, but I don’t know how to talk to them because I was not trained in this material” (Female CHW 476)	“I didn’t have any training. I worked as an assistant at a local health facility…I learned by observing the births that happened and I began to do them as well, in [nurses’] absence” (TBA, 7 years)
±	Supervision		Wanted more supervision to support skill building	Wanted more supervision and accompanying recognition	-	Did not report wanting more supervision
±	Collaboration with other cadres		“There are essential elements for good health in the community, we cannot leave anything out” (F nurse, 3 years). Nurses emphasized importance of TBAs to bring women for facility delivery	Sought to work directly with nurses and with neighbors who wield influence	Worked with TBAs, *activistas*, community leaders in person and via telephone “to harmonize messages” (Male CHW 479)	“It would be important to work with other actors, because that way I could learn from them and I can also teach what I know” (TBA 443)
±	Professional community		Interactions varied by facility leadership, facility-wide initiatives, and staff size	Met weekly for reporting; interacted in the field through supervisory visits or visiting patients in pairs	Met regularly or as-needed basis to coordinate activities, depending on community	Interacted at irregularly held trainings
−	Incorrect/inconsistent IYCF messages across health worker cadres who provide care for HIV-infected mothers					

Abbreviations used include *FGD* focus group discussion, *PO* participant observation, *N/A* not applicable, *ANC* antenatal care, *MCH* maternal and child health

#### Motivation

##### Health worker: intrinsic motivation

Nearly all participants were intrinsically motivated to make a difference in their patients’ and communities’ health (Table 3). HIV-infected *activistas* reported that modeling living healthfully with HIV helped them adhere to their own treatment regimens. CHWs were motivated by their ability to address health concerns in their own households. A few participants across cadres were motivated by continued learning opportunities. All cadres were demotivated by patients not following their recommendations, particularly when this resulted in poor health outcomes.

##### Health worker: extrinsic motivation

All cadres overwhelmingly appreciated recognition from patients, the wider community, and other health workers and were distressed by patient complaints. While the majority of health workers described good inter-cadre relationships, community-based workers were more likely to express concern about how their work was viewed by facility-based workers. Visual identification of roles (e.g., badges t-shirts, hats) was important to un-uniformed facility-based staff, *activistas*, and TBAs.

All cadres were dissatisfied with the current form, amount, or timeliness of compensation. Nearly all community-based workers held another job, predominantly subsistence farming, to support themselves. While one *activista* and one CHW noted they were happy to receive any monetary compensation for work they had previously performed uncompensated, TBAs were uncompensated for referring women for facility-based care but had previously been compensated for assisting in home deliveries (e.g., gifts from mothers, food staples from health facility). *Activistas* and TBAs reported that additional money would help to offset travel costs to and from patients’ homes. However, one highly intrinsically motivated nurse did not believe an increase in remuneration would affect nurse performance:

I don’t believe that increasing our salary would help improve the activities, because if I say this it means that I am accepting that we are holding back care for some women because we receive little [money]. Financial support could be used to help me as an individual, but it is not a way to improve the activities (Female nurse, 7 years of experience).

#### Opportunity

##### Patient: behaviors circumventing care

For nurses, patients not presenting at the health facility, dropping out of care, and not adhering to treatment regimens challenged their ability to deliver PVT care. Patients who gave false addresses (to *activistas*) and called for assistance in already-underway home births (to TBAs) challenged community-based cadres.

##### Work mandate: poor referral systems

Current referral systems were problematic for facility- and community-based cadres. Furthermore, lack of a communication system prevented community-based workers from calling ahead or obtaining transportation for an ill patient or a woman in labor.

##### Work mandate: cumbersome record systems

PVT paper record systems were reported and observed to be cumbersome and, at times, inaccurate. Correct and consistent application of a unique PVT code for each HIV-infected mother and HIV-exposed child dyad was problematic and affected the ability to monitor and evaluate service delivery and patient dropout. Similarly, cumbersome reporting systems challenged *activista* associations’ monitoring and evaluation,

##### Work environment: overburdened

While *activistas*, CHWs, and TBAs generally felt their time commitments were appropriate, nurses frequently reported being overburdened. They served long lines of patients daily and then attended patients who presented for critical care after hours, interfering with nurses’ own childcare.

##### Work environment: supplies

All cadres reported lacking supplies or wanting additional ones to execute their responsibilities. Most problematic was medication stock-outs for PVT. Community-based cadres lamented that they lacked materials to assist in home care (*activistas*), expansion of services to treat adults (CHWs), and kits for attending “emergency” births (TBAs).

##### Work environment: infrastructure

All three health facilities were non-conducive to patient privacy (e.g., lack of screens), two lacked electricity, and one did not have locks or running water. Both *activista* associations lacked their own office space and equipment for report writing.

##### Work environment: distance

Distance was a major challenge to *activistas* and TBAs who traveled to patients’ homes and accompanied them to the health facility. For example, *activistas* were frustrated when they traveled long distances to find the patient was not home.

##### Administrative environment: PVT planning and program coordination

Administrative-level challenges for nurses were tempered by the planned roll-out of new PVT programs, including the “one-stop” strategy to streamline PVT services in mid-2013 and Option B+ to initiate HIV-infected pregnant women on lifelong ART (at the district and large peripheral facility) in 2014. However, at the time of data collection, long waits for CD4 count and PCR infant HIV test results and referral of ART-eligible patients to large facilities were significant challenges to service delivery and retaining women in PVT care. In the community, lack of systematic incorporation of *activistas* and TBAs into the health system resulted in missed opportunities to leverage their motivation and skills.

##### Administrative environment: donors

Discrepancy existed in raises and allowances between positions paid directly by the Ministry of Health and by donors. For example, HIV-specific facility-based workers whose positions were funded through the PEPFAR-implementing partner reported not receiving a raise when other facility-based colleagues did.

#### Ability

##### Health worker: approach to patient interactions

All cadres were sensitive to their patients’ opportunity challenges. Nurses supported women in disclosing their HIV status to their partners and considered maternal finances when counseling on infant- and child-feeding options. Community-based workers were generally very sensitive to maternal poverty, food insecurity, and distance to the health facility.

##### Health worker: knowledge

Although this study was not designed to evaluate knowledge, health workers discussed the infant and young child feeding messages that they counseled women on. All nurses and some community-based health workers relayed the updated guidelines Mozambique had adopted [33], but some *activistas* and TBAs reported outdated and incorrect messages for HIV-infected women. One TBA reported just learning that attending home births without protection was a potential route of HIV exposure.

##### Work environment: training

Nurses, *activistas*, and CHWs received refresher trainings on an annual basis. For nurses, these trainings were provided by the Ministry of Health often at the provincial level. For the *activistas*, training was provided by CARE International, and for CHWs, by the Ministry of Health at the district or facility level. Some TBAs had attended trainings over the decades they had served their communities, which had been provided by different organizations, including CARE, in partnership with the Ministry of Health. All groups reported wanting more training.

##### Work environment: supervision

Nurses, *activistas*, and CHWs welcomed additional supervision to help them learn and to provide on-the-job feedback. In addition, *activistas* wanted supervision from the health facility to increase recognition, tying back to extrinsic motivation.

##### Work environment: professional community

Nurse and CHW interactions within their respective cadres varied according to their health facility and community. *Activistas* met weekly, but TBAs reported interacting with other TBAs at irregularly held trainings.

##### Work environment: collaboration with other cadres

All health workers were open to more collaboration to support mothers through the PVT cascade. Existing intra-cadre collaboration depended on the community leadership and cadre presence in the catchment area.

### Assessing potential for PBIs to address barriers and build on promoters to delivery of PVT services (objective 2)

#### Proposed PBIs by cadre

Maternal and child health nurses and facility-based staff were interested in both personal incentives and reinvestment in service delivery. Suggested individual personal incentives included financial or material rewards such as paid work-related trips to the “best” performing health worker or group. Group incentives that built upon social recognition (e.g., T-shirts and transportation to represent the health facility at district-wide celebrations) were also of interest. Staff proposed reinvesting PBIs in collaborations (e.g., with TBAs, community leaders), social support mechanisms (groups for HIV-infected mothers, fathers of HIV-exposed children), and infrastructural improvements (e.g., increasing patient privacy, attract women to wait ahead of labor) to help retain HIV-infected women and their HIV-exposed children in the cascade of PVT services.

In addition to personal incentives and reinvestment in service delivery, *activistas* were interested in directing incentives to support the sustainability of their associations. *Activistas* proposed using PBIs to build an office for meeting and storage space and to start income-generating activities. Similar to health facilities, *activistas* sought to use incentives (e.g., lunch, transportation) to support knowledge sharing and coordination of care with other community-based actors. *Activistas* saw opportunities to reinvest incentives in service delivery by repairing bicycles, community engagement (e.g., plays, cooking demonstrations), and facilitating community health worker collaborations.

TBAs were primarily interested in PBIs as means of individual financial incentives, materials to support their work (e.g., flashlights, telephone credit, transportation), and collaboration with other community-based cadres.

#### Suggested metrics and concerns for distortions as a result of PBIs

Suggested metrics for awarding incentives at health facilities included evaluation of staff performance through records and patient ratings of the quality of the care interaction. There was interest in both individual and group metrics; however, tracking metrics at the individual health worker level would be difficult with the record systems and reporting practices that were in place. In addition, health facility participants felt that all facility-based workers contributed to clinical PVT care, but tracking PVT indicators would primarily reflect nurse and midwife efforts.

*Activistas* debated whether performance goals should be set at the individual or association level. While all cadres discussed effects on motivation, *activistas* were particularly concerned that awarding PBIs on an individual basis might demotivate their colleagues who did not receive the incentives. *Activistas* were concerned that incentives for particular indicators would detract from their other services.

In contrast to the facility-based workers and *activistas*, TBAs had no interest in setting group goals because they operated individually in their respective communities. TBAs also found individual goal setting problematic because of the variance in number of TBAs and pregnant women in their communities. Like *activistas*, TBAs were concerned with creating competition with their colleagues that would disrupt their collegial relationships.

## Discussion

This theoretically driven evaluation of barriers and promoters of PVT service delivery revealed that health workers were highly motivated but encountered severe opportunity challenges. Facility-based staff were challenged across the ecological motivation-opportunity-ability domains by late payment and uncompensated after-hours work (motivation) and patients circumventing care, poor referral and record systems, heavy workloads, stock-outs, poor infrastructure, and administrative factors (opportunity). We found community-based cadres were dissatisfied with compensation (motivation), challenged by lack of supplies, distance, lack of incorporation into the health system (opportunity), and incorrect and outdated knowledge on infant- and young child feeding (ability).

Our findings reflect challenges encountered by facility-based workers in sub-Saharan Africa [34, 35]. We expand upon structural barriers of absenteeism and irregular supplies experienced by nurses in northern Mozambique [36]. We know that health workers’ dissatisfaction with salary, poor record systems, work overload, and stock-outs can distort service values and feedback into motivation [37, 38]. In contrast, a supportive interpersonal environment (e.g., recognition, mentoring, training) and adequate infrastructure (inclusive of workload, supplies, equipment) are key dimensions of facility-based worker satisfaction [39]. While our study found facility-based workers to be highly motivated, further investigation into workers’ interpersonal environment and barriers’ effects on service values is warranted, including identifying and reducing inefficiencies in workflow [40, 41].

One of the most striking challenges for community-based workers was the lack of integration into the health system, which threatens effective task shifting. Lack of integration and poor follow-up on their referrals prevents community-based providers’ efforts from translating into health impact [42, 43]. In contrast, integrating motivated community-based health cadres can remedy ability and some opportunity barriers [44, 45]. This is particularly salient for TBAs whose knowledge and trainingvaried drastically.

Our data suggest that PBIs would be appropriate for addressing the barriers that most cadres encounter across the ecological levels. PBIs are designed to target workers’ ability to act on intrinsic motivation (by increasing supervision, support, empowerment) and external motivation (through financial and in-kind rewards) and are structured to address opportunity and ability challenges [46]. Facility- and community-based cadres reported that they would leverage PBIs for social recognition (worker level: motivation), patient engagement (patient level: opportunity), referral and record systems (work mandate level: opportunity), infrastructure (work environment: opportunity), and collaboration with other cadres and professional communities (work environment: ability) (Table 4). Indeed, successful PBIs have improved teamwork and record systems [47], enhanced transparency, and reduced corruption [47], all critical aspects of well-functioning heath systems.

**Table 4:** Potential for performance-based incentives (PBIs) to address barriers to health workers’ delivery of prevention of vertical transmission of HIV services by ecological motivation-opportunity-ability factors

Construct	Potential for PBIs
Motivation	
Health worker factors	
Intrinsic motivation	Limited
Extrinsic motivation	Limited
Opportunity	
Patient factors	
Patients circumventing care	Very limited
Work mandate factors	
Referral system	Yes
Record system	Yes
Work environment	
Over-burdened	Limited
Supplies	Limited
Infrastructure	Limited
Distance	Very limited
Administrative environment	
PVT planning and program coordination	Yes
Foreign donors	Unclear
Ability	
Health worker factors	
Health worker approach to patient interactions	Limited
Work environment	
Training	Yes
Supervision	Yes
Collaboration with other cadres	Yes
Professional community	Yes

Our participants’ concerns about the implementation of PBIs align with those in the literature. PBIs can introduce potential for neglect of non-incentivized tasks [48], gaming, and other distortions [49], and perceived unfairness of incentive distributions have led to poor implementation [50, 51]. For these reasons, and our novel finding that collective identity is important to workers in terms of choosing and monitoring incentives, careful creation of indicators and strong monitoring and evaluation systems are necessary to monitor distortions [49]. This focus aligns with implementation science and quality improvement approaches, which hold promise to reduce opportunity barriers across the continuum of PVT care [52] and are necessary to evaluate a well-functioning PBI [53].

We recognize that at the health system scale, incentives cannot overcome major structural barriers [46], including a dearth of qualified human resources with the dedicated time to implement and sustain quality management principles [54, 55]. However, PBIs have successfully improved quality of hospital management and supervision and support for peripheral facilities [56]. Furthermore, sustainability of PBI initiatives, particularly in donor-funded contexts, remains an understudied concern [57]. Thus, we find it appropriate for PBIs to be implemented alongside strong monitoring and evaluation programs to address some challenges faced by health workers delivering PVT services, and we recognize that PBIs cannot replace appropriate health system leadership, policy, and investment.

### Strengths and limitations

We employed a strong qualitative design that led to iterative analysis among the four cadres that deliver PVT services, including often-overlooked TBAs. The use of the ecological motivation-opportunity-ability framework addresses the dearth of multilevel theoretical frameworks that extend beyond an ecological approach to the advanced delivery of PVT care [6]. Finally, we engaged a variety of ground-level stakeholders to assess the context for a PBI intervention, again addressing identified gaps in the literature [58, 59]. A direct outcome of this approach was identifying that TBAs were not comfortable with goal setting, and thus, another approach (e.g., per-service PBI model [60]) may be more appropriate.

This study may not be generalizable to other regions of sub-Saharan Africa due to the history of Mozambique’s health system. However, given similarities in motivation and opportunity challenges across sub-Saharan Africa, this work contributes important perspectives to the appropriateness of a touted method to PVT services. The cross-sectional nature of the interviews may have precluded the building of relationships necessary to discuss grave challenges, particularly with the CHWs and TBAs who were not heavily engaged in participant observation. Participant responses may have been influenced by social desirability bias [61] as well as answering in extremes, since participants knew this research would inform a pilot PBI study with potential for personal financial incentives. Participant TBAs were likely more linked with health facilities than TBAs who did not participate. However, we anticipate this underestimates TBAs’ variance in health facility linkages, and thus, our report on engagement is conservative.

Finally, this paper evaluates the appropriateness of PBIs in addressing barriers that health workers, but not HIV-infected pregnant women and mothers, experience. We recognize that multifaceted and community-engaged solutions are necessary to eliminate the vertical transmission of HIV [62, 63]. However, improving the work environment of facility- and community-based health workers is meritorious in its own right, and improving the delivery of PVT services is one key facet of the solution.

## Conclusions

We found that highly motivated health workers encountered severe opportunity challenges in their mandate to prevent vertical transmission of HIV. PBIs have the potential to address key barriers that facility- and community-based health workers face in delivering care to HIV-infected women and their HIV-exposed children, specifically by building upon existing intrinsic motivation and leveraging highly valued social recognition. Therefore, a controlled intervention monitoring the effects of PBIs on health worker motivation could lead to important insights about the feasibility of PBIs to improve the delivery of PVT services.

## Additional file

Additional file 1: Semi-structured interview guide for four cadres of Mozambican health workers to characterize barriers and promoters to delivering prevention of vertical transmission of HIV services.

## Abbreviations

CHW: Community health worker
PBI: Performance-based incentive
PVT: Prevention of vertical transmission of HIV
TBA: Traditional birth attendant
