# Supplementary material for: Performance-based incentives may be appropriate to address challenges to delivery of prevention of vertical transmission of HIV services in rural Mozambique: a qualitative investigation
Source: Hum Resour Health. 2016 Oct 7;14:60. doi: 10.1186/s12960-016-0157-0 (PMC5054578; doi:10.1186/s12960-016-0157-0)
Supplement: Additional file 1: — Semi-structured interview guide for four cadres of Mozambican health workers to characterize barriers and promoters to delivering prevention of vertical transmission of HIV services. [file 12960_2016_157_MOESM1_ESM.docx]

**I. Work history**

How long have you been working as a [type of health worker]?

How did you become trained in your job?

*When*?

*Who trained you?*

*What sort of training did you receive to do your work?*

*How long was the training?*

Do you still have trainings or updates for your work?

*Can you describe them to me?*

Does provide supervision or support for you?

*Who?*

**II. Responsibilities**

What are all the responsibilities of your work?

How many people do you see in a typical day?

*Does the number of people that you see differ by the season? If yes, how or why?*

When you see patients, could you tell me what a typical workday is like for you?

[FOR COMMUNITY-BASED WORKERS]: How do you identify your patients?

Do patients give you or thank you in some way for the services?

*Why or why not?*

How many weeks or months into their pregnancy are women when you start working with them?

Do you talk with them before their baby is born about getting health care for themselves or their baby after the baby is born?

*What do you say?*

*How do they respond?*

Do you talk with the women after their baby is born about their or the baby’s health?

*What do you say?*

*How do they respond?*

What is your role in preventing vertical transmission of HIV?

What other work do you do that is related to HIV?

Do you also help to care for those who are HIV-infected?

*How*?

Do you ever give advice on getting an HIV test?

*What do you say?*

*How do they respond?*

Do you ever give advice on taking HIV medicines?

*What do you say?*

*How do they respond?*

Do you ever give advice on breastfeeding?

*What do you say?*

*How do they respond?*

*What about exclusive breastfeeding?*

Do you ever give advice on family planning?

*What do you say?*

*How do they respond?*

What challenges do you think women face in accessing the services they need to keep themselves healthy?

*Their babies?*

*Their babies free of HIV?*

*What do you think would help them to get those services?*

Do you have other jobs besides being a [type of health worker]?

*What is it/are they?*

*How much time do you spend per week on your other jobs?*

*Which job do you like best and why?*

**III. Colleagues**

How do you interact with other [type of health worker]?

*When?*

Who are all the other people you work with who also help to prevent vertical transmission of HIV?

*What is your relationship with them?*

*[Probe on]: health facility reception, nurses, community health workers, traditional*

*birth attendants, activistas*

What opportunities do you see to work more closely with any of these groups in keeping women and babies healthy?

*What would help you to do this?*

**IV. Motivation and factors affecting performance**

What led you to begin this work as an ________?

What motivates you to *continue* to do this work?

What do you like about your job?

What do you not like about your job?

When do you feel satisfied in your job?

*Can you give an example or two of a time you felt especially happy and proud in your job?*

When do you feel dissatisfied in your job?

*Can you give an example or two of a time you felt unhappy to be doing your job?*

What makes it difficult to do your work? Why?

*[Probe on]: distance from health center, distance from patients, costs, wages, other responsibilities at home, stigma, time spent*

What would help you to do your work?

*[Probe on]: financial support, supplies, social recognition, additional training, supervision*

**V. Wrap-up questions**

What else should we know about the care of pregnant and recently-delivered women in this region?

What else should we know about the care of women living with HIV in this region?

Do you have any children yourself?

What has been your experience with exclusive breastfeeding?

Do you actively promote exclusive breastfeeding?

What is your personal experience with HIV?
